# Draft Genome Sequence of Chromobacterium violaceum RDN09, Isolated from a Patient with a Wound Infection in Bangladesh

**DOI:** 10.1128/MRA.00957-20

**Published:** 2020-10-15

**Authors:** Razib Mazumder, Ahmed Abdullah, Arif Hussain, Dilruba Ahmed, Dinesh Mondal

**Affiliations:** a Laboratory Sciences and Services Division, International Centre for Diarrhoeal Disease Research, Bangladesh (icddr,b), Dhaka, Bangladesh; Georgia Institute of Technology

## Abstract

Chromobacterium violaceum is an emerging environmental opportunistic pathogen that causes life-threatening infections in humans. Here, we describe the draft genome sequence of Chromobacterium violaceum RDN09, isolated from the infected wound of an adult male patient in Bangladesh. The genome assembly consists of 4,736,739 bp spread across 84 contigs.

## ANNOUNCEMENT

Chromobacterium violaceum is a betaproteobacterium and a member of the family *Neisseriaceae*. It is a saprophyte predominantly present in the natural ecosystems of tropical and subtropical countries of the world (1). This bacterium is known for the production of violacein, a purple-colored pigment produced as a result of quorum sensing (2). Human infections with C. violaceum are rare; however, if contracted, it can cause severe systemic infections with high mortality rates (∼60%) (3). Infections manifest in the form of cellulitis and skin abscesses that can rapidly progress to sepsis, septic shock, and multiple abscesses in the vital organs (4). Its pathogenicity in the mammalian infection model is attributable to the presence of several virulence factors, including two type III secretion systems (T3SSs) (5).

The strain RDN09 was cultured from a pus specimen obtained from an infected wound in the leg of an adult male patient who had suffered an agricultural injury (6). The strain produced a violet pigment and was a Gram-negative coccobacillus (6). Genomic DNA was extracted from a 24-h culture that originated from a single colony of C. violaceum RDN09 grown at 37°C in Luria-Bertani broth, using the QIAamp DNA minikit (Qiagen) and following the Gram-negative bacterial DNA isolation procedure (6). DNA quality was assessed using a NanoDrop spectrophotometer (Thermo Scientific, USA), and quantification was carried out using a Qubit 2.0 fluorimeter (Life Technologies). The sequencing library was prepared from 1 ng genomic DNA using an Illumina Nextera XT DNA library preparation kit as per the manufacturer’s instructions and sequenced on the Illumina NextSeq 500 platform employing the Illumina NextSeq v2.5 reagent kit (2 × 150 bp). Quality checks on the paired-end sequencing reads (150 bp) were performed using FastQC v0.11.11. The genome coverage was found to be 132× by mapping the reads against the reference genome Chromobacterium violaceum ATCC 12472 (7). Trimmomatic v1.01 was used for adapter trimming based on quality scores of Q30 with the following parameters applied: SLIDINGWINDOW:5:15;LEADING:5;TRAILING:5;MINLEN:36;ILLUMINACLIP:path_to_adaptors_sequences/adapter.fasta:2:30:10 (8). *De novo* assembly was conducted using SPAdes v3.11.1 (9). QUAST v5.0.2 was used for quality assessment of the assembly (10). Prokka v1.12 was utilized to annotate the genome with C. violaceum ATCC 12472 as a reference genome (GenBank accession number NC_005085.1) (11). Default parameters were applied for all software unless otherwise mentioned. The genome sequence of C. violaceum strain RDN09 is 4,736,739 bp with a G+C content of 64.83%. It is made up of 84 contigs, having 39 contigs larger than 1,000 bp, and the contig *N*_50_ value is 203,983 bp. A total of 4,316 coding DNA sequences (CDSs) were identified, of which 56% were assigned a putative function, and the remaining CDSs were annotated as hypothetical. We identified 4 rRNA and 83 tRNA sequences in the Chromobacterium violaceum strain RDN09 genome sequence. We were not able to identify any intact prophage regions using the PHASTER algorithm, a new version of PHAST (12). The genome sequence of Chromobacterium violaceum strain RDN09 exhibited 92.97% similarity with C. violaceum strain ATCC 12472 and was found to harbor genetic elements associated with the production of secondary metabolites. This genome sequence showed the presence of a virulence-related type III secretion gene cluster. Unlike the genome of ATCC 12472, which showed the presence of two T3SSs encoded by Cpi-1/-1a and Cpi-2 (5), strain RDN09 showed the presence of only one T3SS encoded by Cpi-1/-1a. The genome sequence described in this article and future comparative genomic studies will be useful in improving our understanding of the biology and pathogenicity of C. violaceum. Such studies will also help decipher the virulence mechanisms and evolution of this emerging pathogen.

### Data availability.

The bacterial whole-genome shotgun project for *Chromobacterium violaceum* RDN09 has been deposited under the BioProject number PRJNA640761, BioSample number SAMN15332061, and SRA accession number PRJNA640761 and in DDBJ/EMBL/GenBank under the accession number JABXOB000000000. The version described in this article is the first version (JABXOB000000000.1).
